# Behavioural and demographic predictors of adherence to three consecutive faecal occult blood test screening opportunities: a population study

**DOI:** 10.1186/1471-2458-14-238

**Published:** 2014-03-07

**Authors:** Amy Duncan, Deborah Turnbull, Carlene Wilson, Joanne M Osborne, Stephen R Cole, Ingrid Flight, Graeme P Young

**Affiliations:** 1 School of Psychology, The University of Adelaide, Adelaide 5005, South Australia, Australia; 2 Cancer Council South Australia, Eastwood 5063, South Australia, Australia; 3 Flinders Centre for Innovation in Cancer, Flinders University, Bedford Park 5042, South Australia, Australia; 4 Bowel Health Service, Repatriation General Hospital, Daw Park 5041, South Australia, Australia; 5 Preventative Health Flagship, Commonwealth Scientific and Industrial Research Organisation (CSIRO), Adelaide 5000, South Australia, Australia

**Keywords:** Colorectal cancer, Faecal occult blood test, Rescreening, Adherence, Psychological factors

## Abstract

**Background:**

Social cognitive variables are often examined for their association with initial participation in colorectal cancer screening. Few studies have examined the association of these variables with adherence to multiple screening offers i.e., rescreening. This study aimed to describe patterns of participatory behaviour after three rounds of screening using faecal immunochemical tests (FIT) and to determine social cognitive, demographic and background variables predictive of variations in adherence.

**Methods:**

Participants were 1,540 men and women aged 50 to 75 living in South Australia who completed a behavioural survey measuring demographic (for example, age, gender) and social cognitive variables relevant to FIT screening (for example, perceived barriers, benefits, self-efficacy). The survey was followed by three, free FIT screening offers mailed on an annual basis from 2008 to 2010. Patterns of participation after three screening rounds were described as one of five screening behaviours; 1) consistent re-participation (adherent with all screening rounds), 2) consistent refusal (adherent with no screening rounds), 3) drop out (adherent with earlier but not later rounds), 4) intermittent re-participation (adherent with alternate rounds) and 5) delayed entry (adherent with later but not initial round(s)). Univariate (Chi Square and Analysis of Variance) and multivariate (Generalised Estimating Equations) analyses were conducted to determine variables predictive of each category of non-adherence (those that did not participate in every screening offer, groups 2, 3, 4 and 5) relative to consistent re-participation.

**Results:**

Significant social cognitive predictors of non-adherence were; less self-efficacy (drop out and consistent refusal), greater perceived barriers (drop out) and lower levels of response efficacy (consistent refusal). Demographic predictors of non-adherence included; male gender (delayed entry), younger age (intermittent, delayed and consistent refusal), less frequent GP visits (intermittent re-participation) and 'ancillary only' private health insurance (drop out). Less satisfaction with screening at baseline predicted drop out, consistent refusal and delayed entry.

**Conclusions:**

Different combinations of demographic and behavioural variables predicted different patterns of rescreening adherence. Rescreening interventions may benefit from a targeted approach that considers the different needs of the population subgroups. Satisfaction with past FOBT screening measured prior to the study screening offers was an important predictor of adherence.

## Background

Adherence to recommendations for screening with faecal occult blood tests (FOBT) has been shown to reduce incidence of, and mortality from, colorectal cancer (CRC) [1]. In Australia, screening using faecal immunochemical tests for haemoglobin (FIT, a type of FOBT) is recommended at least once every two years for those aged 50 and over [2,3]. Screening programs utilising FOB-based tests have been established in many countries including The Netherlands, Italy, the United Kingdom, Australia and France [4,5]. Initial uptake and continued adherence to screening recommendations are crucial to the success of these programs [3,6]. Recent research has highlighted the importance of using longitudinal observations of screening participation to measure adherence adequately [7-11]. Studies of this nature have documented low rates of ongoing adherence ranging from 13.7% [7] to 39.2% [11] for recommended levels of screening participation (i.e., participation in *all* screening offers) over more than two screening rounds. These findings highlight the importance of moving beyond identification of the factors that predict initial uptake to those associated with screening relapse (i.e., program dropout) and other irregular patterns of participation.

The majority of past CRC screening research has focused on predictors of participation in single screening opportunities (i.e., initial uptake or other one-off opportunities) [12-14]. These studies have established the utility of including social cognitive measures to explain initial screening participation and form the basis of many behavioural interventions to improve adherence [12-14]. Our previous work has extended this in order to explore the relationship between social cognitive variables and *intention* to rescreen. Significant predictors of rescreening intention included satisfaction with prior FOBT screening, awareness of the need to repeat screening, greater self-efficacy, greater perceived benefits of screening and fewer perceived barriers [15]. The focus of the current study was to determine predictors of consecutive participation in *more than one* screening opportunity (i.e., repeat or continued adherence) which, from here on, will be referred to as rescreening. Existing research on rescreening has, to date, been primarily limited to an examination of demographic and background (i.e., health systems) factors [9,10,16-19]. Significant positive predictors include greater frequency of general practitioner (GP) visits [17,19], older age [17,18] and male gender [9,17]. However, no studies have examined the utility of social cognitive variables for predicting ongoing adherence.

Recent research has highlighted the importance of utilising descriptive frameworks that encompass multiple participatory patterns of behaviour when measuring, and predicting, rescreening adherence [9,11,18,20]. These frameworks not only allow for the exploration of variables predictive of different non-adherent behaviours (for example, drop out behaviour, consistent non-participation) [9,11,18,20] but they also provide a more accurate measure of ongoing adherence compared to cross sectional data [7]. There is evidence to suggest that interventions targeted to population subgroups are more successful in encouraging participation than a standard population approach [21,22]. Identifying the needs of different non-adherent subgroups could therefore inform the development of interventions to target demographic or behavioural factors that predict various patterns of non-adherent behaviour [9].

The primary aim of this study was to identify factors significantly associated with different categories of adherence defined according to patterns of participation observed after three annual FIT screening offers. Potential social cognitive, demographic and background factors measured at baseline, prior to the first screening offer, and derived from behavioural models that have successfully predicted initial screening uptake [12] were tested for their ability to predict variations in rescreening adherence. The study also aimed to explore the additional contribution of satisfaction with screening, measured prior to the study screening offers, on adherence to patterns of rescreening in the study.

## Methods

### Study population

The study population were 4000 men and women aged 50–75, residing in the surrounding suburbs of Adelaide, South Australia, who were selected at random from data provided by the Australian electoral roll. Elector details were cross checked against a CRC high risk database at the Bowel Health Service (BHS) Repatriation General Hospital in South Australia. Those known to be at high risk for CRC (as identified in the database), defined as having a personal or family history of CRC, or long standing irritable bowel conditions [23], were excluded from possible selection.

### Study design

This study consisted of two phases; the survey phase (baseline) and the screening phase (following baseline). All social cognitive, demographic and background variables utilised in this study were measured in the baseline behavioural survey and used to predict participation in three subsequent screening offers (prospective design).

Participants were mailed a baseline behavioural survey in November of 2008 (survey phase). Those who completed the survey were then invited to participate in free FIT screening coordinated by the BHS on an annual basis for a period of three years (screening phase). In the first instance, survey respondents were mailed the FIT two weeks following return of the survey. Subsequent screening offers were mailed from October 2009 and September 2010, approximately one and two years after the initial offer. Offers were not mailed when 1) an invitee contacted the BHS to opt out of the study or 2) a positive test result in a preceding round, with follow up diagnostic evaluation, precluded the need for further offers. Participants provided informed consent to participate in the study via return of the completed survey and for each screening offer via return of a participant details and consent form and/or return of the completed FIT.

### Materials

#### Behavioural survey

The survey, administered *before* the first screening offer (baseline), was designed to collect information on a variety of social cognitive, demographic and other background variables likely to predict rescreening. This survey has also been described in detail in a paper that reports associations with rescreening intention [15].

##### Demographic variables

Demographic variables included age, gender, education, employment, level of private health insurance coverage and index of relative socioeconomic disadvantage defined according to postcode [24].

##### Social cognitive variables

Social cognitive measures were; barriers and benefits of FOBT screening, perceived CRC susceptibility and severity, level of social influence (from family, friends and health professionals) to screen, a general measure of perceived social support, self-efficacy for completing screening, confidence in FOBT effectiveness (response efficacy), chance health locus of control (belief that health is controlled by chance), internal health locus of control and health value. Social cognitive items (excluding response efficacy which utilised a three point scale with response options of no, unsure and yes) were measured using 5 point Likert scales ranging from strongly disagree to strongly agree. Existing scales were used where possible and example items are provided in Table 1. The measures of perceived barriers and benefits included items from a previous questionnaire designed to measure beliefs regarding initial screening uptake [25] along with new items designed to assess barriers and benefits of rescreening (for example, “having regular home stool tests would give me peace of mind about my health”). A new measure of self-efficacy was also developed for this questionnaire based on the measurement recommendations of Luszczynska and Schwarzer [26].

**Table 1:** Social cognitive items included in the behavioural questionnaire

**Variable**	**Example**	**Items**	**Source**
***Chance health locus of control***	“My good health is largely a matter of good fortune”	4	Wallston et al. [27]
***Internal health locus of control***	“If I take the right actions I can stay healthy”	2	Wallston et al. [27]
***Health value***	“If you don’t have your health you don’t have anything”	4	Lau & Hartman [28]
***Response efficacy***	“Participation in home stool test screening leads to early detection if something is wrong.”	4	Boer & Seydel [29]
***Self-efficacy***	“I am confident that I will be able to screen regularly for bowel cancer with a home stool test even if I find the test to be embarrassing”	6	-
***Barriers***	“Home stool tests are inconvenient”	7	-
***Benefits***	“Screening can pick up bowel cancer early when it can be easily treated”	4	-
***Severity***	“The health consequences of developing bowel cancer are severe”	2	Gregory et al. [25]
***Susceptibility***	“Compared to other people my age my chance of getting bowel cancer is high”	2	Gregory et al. [25]
***Social support***	“I can count on my friends when things go wrong”	6	Gregory et al. [25]
***Social influence***	“My doctor thinks I should have bowel cancer screening”	4	Tiro et al. [30]

##### Background variables

Participants’ knowledge of CRC risk factors (age, family history and lifestyle/diet) and CRC screening (the importance of early detection and treatment, the need to rescreen) were measured using 6 items (example; ‘it is not necessary to screen again for bowel cancer if your previous screening test was normal’) with response options yes, no and don’t know/unsure. Three yes/no items measured participants’ social interactions concerning CRC (known anyone who has had bowel cancer) and screening (discussed home stool testing with anyone, known anyone who has used a home stool test). Other background items included; frequency of general practitioner visits in the year preceding the study (ranging from none to five or more visits) and engagement in other types of cancer screening behaviour (yes or no). Family history of CRC and prior cancer diagnoses (other than CRC) were also measured.

Participants were asked if they had used an FOBT to screen for bowel cancer *prior* to their involvement in the present study (self-report past FOBT use). Those who indicated that they had previously used a FOBT were asked to indicate their overall satisfaction with their past screening experience on a 5 point Likert scale ranging from very unsatisfied to very satisfied.

#### FIT screening offer

Annual screening offers were mailed from the BHS and included; 1) an invitation letter, 2) a two sample immunochemical FIT kit (OC-Sensor, Eiken Chemical Co., Tokyo, Japan), 3) a bowel cancer information brochure, 4) a combined participant details and consent form, 5) a reply-paid envelope and 6) a screening status information form where participants could provide details of participation in other CRC screening tests outside of the study. Reminder letters were mailed to non-participants after six weeks.

### Study outcomes

Screening adherence, determined after three annual FIT screening rounds, was the primary outcome of the study. Adherence per round was measured based on either return of the completed study FIT or self-reported adherence with other CRC screening (for example, colonoscopy or FOBT obtained elsewhere) within the yearly observation period for each offer. Self-report was obtained either via the screening status information form or by participants contacting the BHS directly. Those reporting use of endoscopic screening, (colonoscopy or flexible sigmoidoscopy), with or without study FIT utilisation, were excluded from analyses. This exclusion was because the focus of this study was to determine the relevance of questionnaire variables to the prediction of rescreening adherence using FOB-based screening tests.

Participants were coded as adherent (Y) or non-adherent (N) for each round resulting in eight possible patterns of participation. Participants were first coded according to participation in the study FIT. Additional self-report data for participation in other FOBTs were then included. This process ensured that observed FIT participation was the main source of data, which was then supplemented with self-report data.

#### Defining patterns of participatory behaviour

The eight possible patterns of participation were collapsed into five categories of adherence behaviour based on those described by Cole et al. [9]. The categories were; consistent re-participation (adherent with all screening rounds; target behaviour), consistent refusal (adherent with no screening rounds), drop out (participation round one and/or round two followed by subsequent refusal) and delayed entry (initial refusal followed by participation in later rounds). An additional behavioural category, intermittent re-participation, was also included in order to describe those who dropped out in round two but then re-participated in the final screening round. The eight participation patterns and associated adherence categories are described in Table 2.

**Table 2:** FIT screening patterns and behaviours defined after three annual screening rounds

**Participation patterns across three screening rounds**			**Adherence category**
***Round 1***	***Round 2***	***Round 3***	
Y	Y	Y	Consistent re-participation
Y	Y	N	Drop out
Y	N	N	
N	Y	N	
Y	N	Y	Intermittent re-participation
N	Y	Y	Delayed entry
N	N	Y	
N	N	N	Consistent refusal

#### Analyses

##### Primary analyses

Univariate (ANOVA and Chi squared) comparisons with post hoc tests (Hochberg GT2 and Chi Square with a Bonferroni correction) were conducted comparing consistent re-participation with each remaining category (the ‘non-adherent’ categories; drop out, intermittent re-participation, delayed entry, consistent refusal) for each of the demographic, background and social cognitive variables. Skewed variables were analysed using non-parametric alternatives [31]. Significant differences determined at the univariate level were then incorporated into four separate multivariate models, utilising log Poisson Generalised Estimating Equations (GEE), with consistent re-participation as the referent category.

##### Secondary analyses

To determine the impact of satisfaction with screening *before* study involvement on adherence to screening offered during the study, a second multivariate analysis was conducted on the subpopulation of participants who indicated in the baseline survey that they had used an FOBT in the past (self-reported past FOBT use). These models incorporated all the variables included in the primary multivariate analyses with the addition of a single item measure of satisfaction with past screening.

### Exclusions

**Figure 1:**
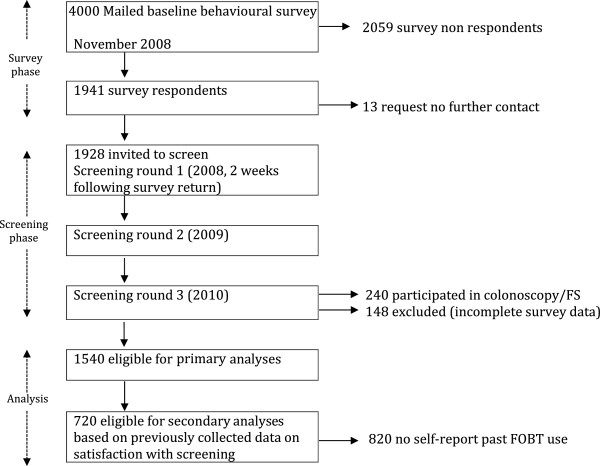
Process of determining eligibility for analyses.

Questionnaire response rate was 48.5% (1941/4000). Thirteen respondents requested no further contact following questionnaire completion therefore annual screening was offered to the remaining 1928 respondents. Screening invitees who participated in endoscopic screening (colonoscopy/FS; n = 240, 12.4%) and those with incomplete questionnaire data (n = 148, 7.6%) were excluded from analyses. The remaining 1,540 questionnaire respondents (79.3%) were included in the analyses for this study as outlined in Figure 1.

### Ethical considerations

This study has been approved by the Human Research Ethics Committees at the University of Adelaide and the Repatriation General Hospital.

## Results

### Description of the sample

The final sample (n = 1540) comprised 710 (46.1%) men and 830 (53.9%) women aged 50–75 (mean = 59.94, SD = 6.48). Of the study sample 1,412 (91.75%) participated only in the FIT offered by the BHS whilst the remaining 128 (8.25%) reported other FOBT participation either in addition to, or instead of, the screening offered by the BHS. The majority were married (n = 1189, 77.2%), had completed at least secondary school education (n = 1041, 67.6%), spoke English at home (n = 1329, 86.3%), were born in Australia (n = 1094, 71.0%), and just over half were still in the workforce (n = 802, 52.1%). Index of relative disadvantage scores were divided into quintiles with the lowest quintile (1) indicating greater levels of disadvantage and the highest (5) indicating lower levels of disadvantage. A substantial portion of participants were in the highest (n =453, 29.4%) and lowest quintiles (n = 326, 21.2%) with the remainder spread approximately evenly between. These scores indicate that participants were from a broad range of socioeconomic backgrounds with slightly larger clusters at each extreme. Compared to survey non respondents, survey respondents were more likely to be from less disadvantaged areas (*χ*^*2*^ (4) = 68.36, p < .001) and were slightly less likely to be from the youngest (50–54, 55–59) and oldest (70–74) age groups (*χ*^*2*^ (5) = 24.92, p < .001).

### Rescreening adherence

Table 3 shows the proportion of participants for each category of adherence. Just over 55% of invitees were adherent with annual screening after three years.

**Table 3:** Proportion of study participants in each adherence category upon study completion (i.e., at year 3)

**Adherence category**	**Number (%)**
Consistent re-participation	860 (55.8%)
Drop out	134 (8.7%)
Intermittent re-participation	68 (4.4%)
Delayed entry	166 (10.8%)
Consistent refusal	312 (20.3%)
Total	1540 (100%)

### Univariate differences between consistent re-participation and non-adherence

Table 4 shows the significant univariate predictors of categories of non-adherence compared to consistent re-participation. Significant post hoc comparisons, indicated with asterisks, highlight differences between consistent re-participation and each of the non-adherent categories. Social cognitive variables only differentiated consistent re-participation from drop out and consistent refusal. Demographic predictors were identified for all behavioural categories as shown in Table 5.

**Table 4:** Significant social cognitive differences between consistent re-participation and each non-adherent category

**Variable**	**Consistent re-participation (n = 860)**	**Drop out (n = 134)**	**Intermittent re-participation (n = 68)**	**Delayed entry (n = 166)**	**Consistent refusal (n = 312)**	**F(df)**	**p**
	***Mean (SD)***						
*Barriers*	*16.28 (4.22)*	17.96 (4.33)**	16.51 (3.65)	16.57 (4.34)	18.04(4.80)**	12.46 (4,709.51)	<.001
*Benefits*	*16.14 (1.96)*	15.74 (1.99)	15.96 (1.75)	15.90 (1.93)	15.07(2.24)**	16.49 (4,1535)	<.001
*Self-efficacy*	*23.73 (4.69)*	22.36 (4.29)*	23.63 (4.22)	23.35 (4.61)	21.57(5.01)**	14.24 (4,678.72)	<.001
*Response efficacy*	*11.06 (1.45)*	11.26 (1.13)	10.79 (1.88)	10.96 (1.61)	10.46(1.73)**	36.94 (4)^a^	<.001
*Social support*	*22.96 (3.81)*	22.91 (3.61)	22.88 (3.26)	22.47 (3.52)	21.80(4.38)**	5.80 (4,725.81)	<.001
*Social influence*	*13.76 (2.60)*	13.52 (2.61)	13.13 (2.65)	13.38 (2.43)	12.75(2.78)**	8.79 (4,1535)	<.001

*Note*. F = F ratio; df = degrees of freedom, SD = standard deviation, p = probability. Data were measured on increasing, continuous scales.

^a^data were not normally distributed therefore the Kruskal-Wallis test was reported.

*p < .05. **p < .01.

**Table 5:** Demographic and background differences between consistent re-participation and categories of non-adherence

**Variable**	**Consistent re-participation (n = 860)**	**Drop out (n = 134)**	**Intermittent re-participation (n = 68)**	**Delayed entry (n = 166)**	**Consistent refusal (n = 312)**	**F (df)**	**p**
***Mean(SD)***							
Age^a^	*61.18 (6.33)*	58.86 (6.55)**	57.69 (6.20)**	57.86 (5.57)**	58.57 (6.62)**	19.92 (4,599.32)	<.001
*GP visits*^*a*^	*3.98 (1.16)*	4.04 (1.13)	3.47 (1.31)**	3.95 (1.23)	3.85 (1.29)	3.29 (4,546.14)	.011
***Number (%)***						***χ***^***2 ***^**(df)**	**p**
Private health insurance, *None*	*145 (17.0)*	33 (25.0)**	17 (25.0)	31 (18.7)	75 (24.1)	26.80 (12)	.008
*Ancillary*	*74 (8.7)*	21 (15.9)	6 (8.8)	9 (5.4)	21 (6.8)		
*Hospital*	*55 (6.4)*	5 (3.8)	4 (5.9)	9 (5.4)	14 (4.5)		
*Both*	*579 (67.9)*	73 (55.3)	41 (60.3)	117 (70.5)	201 (64.6)		
Gender (*male)*	*369 (42.9)*	58 (43.3)	24 (35.3)	100 (60.2)**	159 (51.0)	23.48 (4)	<.001
Married (*yes)*^*b*^	*689 (80.7)*	93 (70.5) **	54 (79.4)	131 (79.4)	222 (71.2)**	16.46 (4)	.002
Workforce *(*yes)^b^	*383 (45.4)*	78 (60.0)**	43 (64.2)**	109 (66.5)**	189 (61.4)**	45.88 (4)	<.001
Disadvantage, 1	*167 (19.4)*	28 (20.9)	16 (23.9)	34 (19.4)	81 (26.0)**	36.73 (16)	.002
*2*	*140 (16.3)*	35 (26.1)	11 (16.4)	26 (15.8)	56 (18.0)		
*3*	*152 (17.7)*	20 (14.9)	16 (23.9)	20 (12.1)	39 (12.5)		
*4*	*149 (17.3)*	24 (17.9)	4 (6.0)	31 (18.8)	34 (10.9)		
*5 (least)*	*251 (29.2)*	27 (20.1)	20 (29.9)	54 (32.7)	101 (32.5)		
Knowledge (*incorrect)*^*c*^	*124 (14.5)*	23 (17.2)	13 (19.4)	23 (13.9)	72 (23.1)**	13.51 (4)	.009
Known CRC (*yes*)^*b*^	*622 (72.7)*	83 (62.9)	43 (63.2)	105 (63.6)	191 (62.0)**	17.40 (4)	.002
Discussed FOBT (*yes)*^*b*^	*542 (63.0)*	65 (48.9)**	39 (57.4)	91 (55.2)	120 (38.7)**	57.59 (4)	<.001
Known a screener (*yes)*^*b*^	*469 (54.7)*	54 (40.6)**	33 (48.5)	63 (38.2)**	93 (30.1)**	63.00 (4)	<.001
Past other screening (*yes)*^*b*^	*564 (65.9)*	80 (60.2)	39 (57.4)	116 (70.3)	174 (56.1)**	14.42 (4)	.006
SR past FOBT (yes)^b^	*482 (56.0)*	45 (33.6)**	32 (47.1)	83 (50.0)	78 (25.0)**	99.19 (4)	<.001

*Note. SD = standard deviation; F = F ratio; χ*^*2*^ *= chi square; p = probability; SES = socioeconomic status determined by SEIFA index of disadvantage; knowledge = response to knowledge item regarding the importance of repeated screening; Known CRC = known a person who has had CRC; Discussed FOBT = ever discussed FOBT screening; Known a screener = known a person who has screened for CRC; Other screening = ever participated in other (not CRC) screening tests. SR past FOBT = self-reported past FOBT use .*^*a*^*variables were measured on increasing, continuous scales;*^*b*^*comparative category is ‘no’;*^*c*^comparative category is ‘correct’ *p < .05, ** p < .01.

### Multivariate predictors of non-adherence (primary analyses)

Table 6 presents the results of four separate multivariate models with consistent re-participation as the referent category for each. Risk ratios (RR) above 1 indicate an increase in the likelihood of being in the comparative non-adherent category compared with consistent re-participation, and a value below 1 indicates a decrease in likelihood. All social cognitive variables were measured on an increasing scale. Only variables that were identified post hoc as being associated with each category were entered into the multivariate models, therefore each model varies in terms of potential predictors. Significant multivariate predictors are highlighted in bold.

**Table 6:** Multivariate predictors of non-adherence relative to consistent re-participation

**Variable**		**Drop out n = 129**^**a**^			**Intermittent re-participation n = 67**^**a**^			**Delayed entry n = 163**^**a**^			**Consistent refusal n = 300**^**a**^		
		**RR**	**p**	**95% CI**	**RR**	**p**	**95% CI**	**RR**	**p**	**95% CI**	**RR**	**p**	**95% CI**
*Social cognitive*	*Barriers*	**1.05**	**.032**	**(1.00-1.10)**							1.01	.620	(0.98-1.03)
	*Benefits*										0.97	.231	(0.91-1.02)
	*Self-efficacy*	**0.97**	**.047**	**(0.94-1.00)**							**0.97**	**.004**	**(0.95-0.99)**
	*Response efficacy*										**0.93**	**.004**	**(0.88-0.98)**
	*Social support*										1.01	.452	(0.99-1.04)
	*Social influence*										0.97	.281	(0.93-1.02)
*Background*	*Known CRC (yes)*^*b*^										0.88	.173	(0.73-1.06)
	*Discussed FOBT (yes)*^*b*^	1.02	.917	(0.71-1.47)							0.97	.806	(0.77-1.23)
	*Known a screener (yes)*^*b*^	0.86	.390	(0.60-1.22)				**0.72**	**.029**	**(0.53-0.97)**	**0.73**	**.010**	**(0.58-0.93)**
	*GP visits*				**0.80**	**.015**	**(0.67-0.96)**						
	*Knowledge (incorrect)*^*c*^										1.07	.573	(0.85-1.33)
	*Other screening (yes)*^*b*^										0.88	.168	(0.73-1.06)
	*SR past FOBT (yes)*^*b*^	**0.66**	**.042**	**0.44-0.98**							**0.59**	**<.001**	**(0.45-0.77)**
*Demographic*	*Age*	0.98	.157	(0.94-1.01)	**0.93**	**.007**	**(0.88-0.98)**	**0.94**	**<.001**	**(0.91-0.97)**	**0.98**	**.011**	**(0.96-0.99)**
	*Workforce (yes)*^*b*^	1.38	.116	(0.93-2.05)	1.05	.869	(0.60-1.82)	1.21	.302	(0.84-1.75)	1.25	.064	(0.99-1.57)
	*Married (yes)*^*b*^	0.77	.133	(0.54-1.09)							0.82	.068	(0.67-1.02)
	*Private insurance (none)*^*d*^	1.43	.076	(0.96-2.13)									
	*Private insurance (ancillary only)*^*d*^	**1.57**	**.044**	**(1.01-2.43)**									
	*Private insurance (hospital only)*^*d*^	0.75	.501	(0.33-1.73)									
	*Gender (male)*^*e*^							**1.77**	**<.001**	**(1.32-2.38)**			
	*Disadvantage (1)*										0.99	.926	(0.78-1.25)
	*Disadvantage (2)*										0.87	.312	(0.66-1.14)
	*Disadvantage (3)*										**0.71**	**.031**	**(0.53-0.97)**
	*Disadvantage (4)*^*f*^										0.76	.098	(0.55-1.05)

*Note.* RR = risk ratio; p = probability; significance level is p<.05, significant values in bold; 95% CI = confidence interval. Social cognitive variables, GP visits and age were measured on increasing, continuous scales.

*knowledge = response to knowledge item regarding the importance of repeated screening; Known CRC = known a person who has had CRC; Discussed FOBT = ever discussed FOBT screening; Known a screener = known a person who has screened for CRC; Other screening = ever participated in other (not CRC) screening tests; SR past FOBT = self-reported past FOBT use*^*a*^*Cases with missing data were removed list wise, group totals reported in the table report the number of participants included in each analysis.*^*b*^*Reference category is ‘no’*^*c*^*reference category is ‘correct’*^*d*^*reference category is ‘hospital and ancillary combined cover’;*^*e*^*Reference category is ‘female’;*^*f*^*Reference category is the fifth quintile of disadvantage (least disadvantage).*

Three social cognitive variables significantly predicted variations in adherence; a perception of more extensive barriers and lower confidence in utilising the FIT (i.e., less self-efficacy) predicted drop out behaviour. Lower confidence in both personal capacity (i.e., self-efficacy) and test effectiveness (i.e., response efficacy) significantly predicted consistent refusal although the risk ratios were small.

Several demographic and background variables predicted non-adherence to rescreening. Significant predictors of drop out were; having 'ancillary' only private health insurance cover and no past FOBT use ^a^. Younger age and fewer GP visits in the year preceding the study predicted intermittent re-participation, whilst those who entered the screening program later (delayed entry) were more likely to be male and younger, and less likely to have known someone who has screened with a FOBT. Those who consistently refused the screening offers were less likely to be from areas of moderate disadvantage, were younger, had not used a FOBT in the past and were less likely to have known someone else who had used a FOBT.

### Satisfaction with past FOBT use (secondary analyses)

A measure of satisfaction was only available for participants who reported in the baseline questionnaire that they had participated in FOBT screening before being invited into the present study (self-reported past FOBT use, n = 720). Among this subsample there were substantially more participants in the consistent re-participation category (n = 482, 66.9%) and substantially less in the consistent refusal category (n =78, 10.8%) when compared to the full sample. Proportions of those in the delayed entry (n = 83, 11.5%), intermittent re-participation (n = 32, 4.4%), and drop out (n = 45, 6.3%) categories remained relatively similar in the subpopulation.

Details of past screening were limited to a single item measure of overall satisfaction with the screening experience. To determine the potential impact of satisfaction with past screening on non-adherence to the screening offered in this study the multivariate models described previously (primary analyses) were repeated for the reduced sample with the addition of the satisfaction measure as shown in Table 7. Contrary to the primary analyses, social cognitive variables did not differentiate categories of non-adherence amongst a sample of participants who had previously participated in FOBT screening when satisfaction with past screening was taken into account. However, less satisfaction with past screening, was significantly predictive of all non-adherent behaviours with the exception of intermittent re-participation.

**Table 7:** Multivariate predictors of non-adherence relative to consistent re-participation including a measure of prior satisfaction with FOBT

**Variable**		**Drop out n = 40**^**a**^			**Intermittent re-participation n = 31**^**a**^			**Delayed entry n = 81**^**a**^			**Consistent refusal n = 76**^**a**^		
		**RR**	**p**	**95% CI**	**RR**	**p**	**95% CI**	**RR**	**p**	**95% CI**	**RR**	**p**	**95% CI**
*Behavioural (social cognitive and satisfaction)*	*Barriers*	1.07	.112	(0.98-1.17)							1.00	.873	(0.93-1.06)
	*Benefits*										1.01	.936	(0.89-1.14)
	*Self-efficacy*	1.00	.965	(0.94-1.17)							1.00	.847	(0.96-1.05)
	*Response efficacy*										0.94	.333	(0.82-1.07)
	*Social support*										1.02	.455	(0.97-1.08)
	*Social influence*										0.94	.186	(0.86-1.03)
	*Satisfaction*	**0.60**	**<.001**	**(0.46-0.78)**	0.99	.960	(0.66-1.48)	**0.78**	**.008**	**(0.65-0.94)**	**0.74**	**.005**	**(0.60-0.92)**
*Background*	*Known CRC (yes)*^*b*^										0.80	.348	(0.51-1.27)
	*Discussed FOBT (yes)*^*b*^	0.84	.642	(0.40-1.75)							0.82	.465	(0.50-1.37)
	*Known screener (yes)*^*b*^	1.20	.582	(0.62-2.31)				0.76	.183	(0.51-1.14)	0.74	.153	(0.49-1.11)
	*GP visits*				**0.64**	**<.001**	**(0.51-0.82)**						
	*knowledge (incorrect)*^*c*^										1.27	.458	(0.67-2.40)
	*Other screening (yes)*^*b*^										0.97	.891	(0.63-1.50)
*Demographic*	*Age*	0.99	.654	(0.93-1.05)	0.96	.218	(0.89-1.03)	**0.93**	**<.001**	**(0.89-0.97)**	0.97	.209	(0.93-1.02)
	*Workforce (yes)*^*b*^	1.92	.067	(0.95-3.84)	1.49	.256	(0.75-3.00)	1.41	.198	(0.84-2.39)	**1.73**	.054	**(0.99-3.03)**
	*Married (yes)*^*b*^	0.95	.896	(0.45-1.95)							0.98	.918	(0.61-1.57)
	*Gender (male)*^*d*^							**2.00**	**.002**	**(1.30-3.10)**			
	*Disadvantage (1)*										**0.49**	**.050**	**(0.24-1.00)**
	*Disadvantage (2)*										0.66	.155	(0.37-1.17)
	*Disadvantage (3)*										**0.41**	**.023**	**(0.19-0.88)**
	*Disadvantage (4)*^*e*^										**0.48**	**.032**	**(0.25-0.94)**

*Note.* RR= risk ratio; p= probability, 95% CI= confidence interval.Social cognitive variables, satisfaction, GP visits and age were measured on increasing, continuous scales. Health insurance was not included as a predictor in secondary analyses as cell sizes were <5 for drop out participants with only ancillary and only hospital cover.

knowledge= response to knowledge item regarding the importance of repeated screening; Known CRC= known a person who has had CRC; Discussed FOBT= ever discussed FOBT screening; Known a screener= known a person who has screened for CRC; Other screening= ever participated in other (not CRC) screening tests. ^a^ Cases with missing data were removed list wise, group totals reported in the table report the number of participants included in each analysis. ^b^ Reference category is ‘no’ ^c^ reference category is ‘correct’; ^d^ Reference category is ‘female’; ^e^ Reference category is the fifth quintile of disadvantage (least disadvantage).

## Discussion

Recent studies have highlighted the importance of monitoring longitudinal screening adherence in order to measure rescreening accurately [7,9,11]. In this study, ongoing adherence was measured according to a predefined framework that described a variety of screening behaviour patterns over a period of three years [9]. Results show just over 55% of participants provided with annual invitations to screen with FIT were adherent with all three rounds. A substantial portion of participants were non-responsive to all invitations (20.3%), and irregular patterns of adherence were observed for the remaining 24%.

The substantial portion of those who participated at irregular intervals, or refused screening altogether, over a three year time frame highlight the importance of identifying ways to encourage ongoing adherence in order to maximise the population benefit of FOB-based screening programs [3,11]. Furthermore, results found different demographic and behavioural variables to be predictive of different patterns of adherence over the three year observation period. These findings support the hypothesis that different types of non-adherent behaviours are likely to respond to different types of interventions [9].

Identifying demographic sub groups less likely to respond to ongoing screening opportunities can assist with the design of targeted interventions to improve adherence [9]. Some demographic predictors of rescreening (for example, older age [17]) have already been identified. This study is the first to examine predictors of adherence after more than two screening rounds, and included a broad range of demographic variables, which led to some new findings. For example, results showed that men were more likely to delay participation in the screening program. Prior research has consistently reported lower levels of initial FOBT participation amongst men [32-34]. However these findings suggest that men may simply take longer to respond to invitations to screen, and may benefit from different recruitment strategies than women to encourage first time compliance. Similarly, having private health insurance for ancillary services only was predictive of screening drop out but not associated with other behaviours including consistent non-participation. Results may reflect concerns about ongoing costs resulting from follow up testing in initial screening rounds [35]. Screening programs may want to consider finding means of providing free follow up examinations to encourage rescreening.

Consistent with our previous research on rescreening intention [15], satisfaction with past FOBT screening was a strong behavioural predictor of adherence to the study FIT offers. Satisfaction was one of the few variables to predict multiple screening behaviours with less satisfaction with past screening predicting likelihood of drop out, consistent refusal and delayed entry behaviour by 40%, 26% and 22% respectively. The mammography screening literature has consistently shown less satisfaction with initial screening to be predictive of non-adherence to a subsequent offer [36,37]. Results of the present study indicate that satisfaction can also have an ongoing effect on adherence leading to consistent refusal of multiple screening offers as well as irregular participatory patterns. The importance of ensuring initial and continued satisfaction with screening cannot be underestimated. Future rescreening research should consider exploring which aspects of screening, for example, the type of FOB test utilised [38], the service provided as part of the screening experience [37] or the receipt of abnormal test results [16] may contribute to perceived satisfaction and how these factors may be improved to encourage rescreening.

A review of the screening literature where faecal occult blood tests have been used indicates that social cognitive variables can predict initial screening uptake [12] and rescreening intention [15]. In the present study, greater perceived barriers and less self-efficacy predicted drop out behaviour whilst less self-efficacy and less confidence in the efficacy of the test (response efficacy) predicted consistent non-participation. Social cognitive variables did not predict either delayed entry or inconsistent re-participation. Results indicate that interventions targeting relevant beliefs, for example those that aim to reduce perceived barriers to screening, emphasise the utility of FOBT for early detection (e.g., improve perceived effectiveness and utility of the screening test), and highlight ease and simplicity of testing (e.g., improve confidence in personal ability to complete the test), may improve rescreening adherence for subgroups of the population.

The relationship between greater perceived barriers, lower self-efficacy and non-adherence to initial screening has been well established [12,13]. The finding that consistent refusal of screening in this study was associated with lower *response* efficacy for FOBT screening is an interesting contribution. Whilst several qualitative studies have highlighted participant concerns about the efficacy of home stool tests [35,39] few quantitative investigations have established a relationship between these beliefs and initial participation. The results of the present study may indicate that the behaviour of refusing a single screening offer is different from the behaviour of refusing multiple screening offers. Consistent refusal might instead reflect an informed decision (the decision to refuse screening is based on adequate knowledge of the screening test, its purpose and limitations [40]) to not participate in *FOB-based* CRC screening. Further research is required to establish whether lower response efficacy for FOBT in this study was a result of inadequate knowledge of FOBT or reflects a preference for alternate forms of screening such as colonoscopy.

Whilst this study identified several social cognitive predictors of patterns of adherence, the risk ratios associated with these predictors were smaller than those of similar studies in the initial screening literature [25,41]. There are several possible explanations for this finding.

Firstly, this study utilised a prospective design where beliefs about FOBT screening were obtained before the study outcomes (participation in three rounds of screening) were measured. Results presented here therefore cannot account for possible changes in these beliefs that may have occurred over the three year screening period [42]. Secondly, whilst the study utilised existing social cognitive scales where possible, some variables (for example, severity) were measured using only two items, whilst satisfaction was measured with only one item. It may be necessary to include more extensive measures in the future in order to increase the reliability of the results, particularly in relation to satisfaction. Results may also indicate that additional psychosocial variables are required to explain adherence behaviour. Future research should consider exploring the use of other behavioural variables, for example those associated with behaviour *maintenance* (for example, planning processes and maintenance self-efficacy [43]), to better explain variations in rescreening adherence.

Finally, the small sample sizes of some adherence categories (for example the drop out and intermittent reparticipation categories), particularly in the secondary analyses, should also be taken into consideration. Ideally, for multivariate analyses, the number of parameters included in each model should be n/10- 1 where n is the sample size of the smallest comparison group [44]. Whilst these requirements were met in the primary analyses, the group sizes were too small to meet these requirements amongst the satisfaction subgroup analyses. It is possible therefore that the contribution of the social cognitive variables were small or non-existent as a result of the small sample size rather than a lack of association. Repeating the analyses with a larger sample, or collapsing some of the adherence categories, may be required to establish the contribution of social cognitive variables to adherence behaviour particularly when satisfaction with prior screening is taken into consideration.

This study is amongst the first to document patterns of ongoing adherence to FOBT screening [9,11]. In this study, outcomes were based on both observed (per protocol) and observed plus self-reported (per and ex protocol) adherence to annual FOB-based screening. The inclusion of self-report data ensured that participants were not misclassified as being non-adherent when screening had occurred independent of the study [45]. This is particularly important in the current Australian context where a national bowel cancer screening program offers free screening to select age groups within the target population [3,5] and people can also access tests from doctors and pharmacies. Self-report data however is limited by inaccurate recall [46] and the potential for participants to exaggerate compliance to conform with study or personal expectations [47]. Whilst efforts were made to simplify self-report by restricting recall to a single recent screening experience [48,49], results should be interpreted in light of these potential limitations. In addition, this study measured adherence to an *annual* program of FIT screening. Australian screening recommendations allow for either an annual or beiannial screening interval [2] and it is therefore possible that some participants participated irregularly as a result of these recommendations.

This study is the first to identify differences in behavioural and demographic characteristics of non-adherence beyond two screening rounds. Whilst observed differences between the adherence categories for demographic, background and social cognitive variables support the use of a descriptive framework for understanding rescreening adherence there are limitations to this approach.

Screening participation is an ongoing behaviour; the analyses presented here describe participants based on observed behaviour only over three consecutive screening rounds, and labels assigned to participants’ behaviours will change from one round to the next; category membership is not fixed. The findings presented here suggest characteristics that may be targeted at a broad population level to improve rates of participation; but they may not be useful for determining how an individual may behave in the future.

In addition, predefined behavioural definitions were used to guide the categorisation of invitees and some definitions collapsed multiple patterns of behaviour. This approach was beneficial as it allowed for a detailed comparison of several different types of adherence behaviour without focusing on all eight of the observed patterns. This is particularly relevant for studies focusing on greater numbers of screening rounds, where possible patterns of behaviour will continue to increase. This approach does however overlook the potential for differences *within* the categories, for example, potential differences between sustained and sporadic participation [9]. Future research may want to consider exploring alternate rules for categorisation in order to determine the most effective approach for describing, and improving, rescreening adherence.

## Conclusions

This study identified several demographic, background and behavioural variables predictive of non-adherence to three annual offers of FIT screening for CRC. Different combinations of demographic and behavioural variables predicted different patterns of rescreening adherence. Less satisfactory experiences with screening before study involvement was a major determinant of non-adherence to the screening offered in the study.

## Endnote

^a^‘Ancillary only’ is a private health insurance policy available in Australia that provides benefits for ancillary treatments (e.g., dental, physiotherapy) but does not provide benefits for hospital visits.

## Abbreviations

ANOVA: Analysis of variance; BHS: Bowel health service; CRC: Colorectal cancer; FIT: Faecal immunochemical test; FOBT: Faecal occult blood test; FS: Flexible sigmoidoscopy; GEE: Generalised estimating equations; GP: General practitioner; RR: Risk ratios.

## Competing interests

All authors declare that they have no competing interests.

## Authors’ contributions

AD contributed to the design of the study and the survey materials, was involved in participant recruitment and data collection, conducted the analyses and drafted the manuscript. DT and CW were responsible for the conception and design of the study, contributed to the development of survey materials, assisted with interpretation of analyses and made substantial contributions to the drafting of the manuscript. JO was involved in participant recruitment, data collection, initial analyses and contributed to the drafting of the manuscript. SC, IF and GP were responsible for the conception and design of the study, contributed to the development of study materials and to the drafting of the manuscript. All authors read and approved the final manuscript.

## Pre-publication history

The pre-publication history for this paper can be accessed here:

http://www.biomedcentral.com/1471-2458/14/238/prepub
